# The Relative Abundances of Human Leukocyte Antigen-E, α-Galactosidase A and α-Gal Antigenic Determinants Are Biased by Trichostatin A-Dependent Epigenetic Transformation of Triple-Transgenic Pig-Derived Dermal Fibroblast Cells

**DOI:** 10.3390/ijms231810296

**Published:** 2022-09-07

**Authors:** Marcin Samiec, Jerzy Wiater, Kamil Wartalski, Maria Skrzyszowska, Monika Trzcińska, Daniel Lipiński, Jacek Jura, Zdzisław Smorąg, Ryszard Słomski, Małgorzata Duda

**Affiliations:** 1 Department of Reproductive Biotechnology and Cryoconservation, National Research Institute of Animal Production, Krakowska 1 Street, 32-083 Balice, Poland; 2 Department of Histology, Jagiellonian University Medical College, Kopernika 7 Street, 31-034 Kraków, Poland; 3 Department of Biochemistry and Biotechnology, Poznań University of Life Sciences, Dojazd 11 Street, 60-647 Poznań, Poland; 4 Institute of Human Genetics, Polish Academy of Sciences, Strzeszyńska 32 Street, 60-479 Poznań, Poland; 5 Department of Endocrinology, Institute of Zoology and Biomedical Research, Faculty of Biology, Jagiellonian University in Krakow, Gronostajowa 9 Street, 30-387 Kraków, Poland

**Keywords:** swine, trichostatin A, epigenetic transformation, ex vivo model, tri-genetically modified, ACFC line, HLA-E, rhα-Gal A, rhα1,2-FT, α-Gal antigenic determinant, porcine skin xenograft

## Abstract

The present study sought to establish the mitotically stable adult cutaneous fibroblast cell (ACFC) lines stemming from h*FUT2*×h*GLA*×*HLA-E* triple-transgenic pigs followed by trichostatin A (TSA)-assisted epigenetically modulating the reprogrammability of the transgenes permanently incorporated into the host genome and subsequent comprehensive analysis of molecular signatures related to proteomically profiling the generated ACFC lines. The results of Western blot and immunofluorescence analyses have proved that the profiles of relative abundance (RA) noticed for both recombinant human α-galactosidase A (rhα-Gal A) and human leukocyte antigen-E (HLA-E) underwent significant upregulations in tri-transgenic (3×TG) ACFCs subjected to TSA-mediated epigenetic transformation as compared to not only their TSA-unexposed counterparts but also TSA-treated and untreated non-transgenic (nTG) cells. The RT-qPCR-based analysis of porcine tri-genetically engineered ACFCs revealed stable expression of mRNA fractions transcribed from h*FUT2*, h*GLA* and *HLA-E* transgenes as compared to a lack of such transcriptional activities in non-transgenic ACFC variants. Furthermore, although TSA-based epigenomic modulation has given rise to a remarkable increase in the expression levels of Galα1→3Gal (α-Gal) epitopes that have been determined by lectin blotting analysis, their semi-quantitative profiles have dwindled profoundly in both TSA-exposed and unexposed 3×TG ACFCs as compared to their nTG counterparts. In conclusion, thoroughly exploring proteomic signatures in such epigenetically modulated ex vivo models devised on h*FUT2*×h*GLA*×*HLA-E* triple-transgenic ACFCs that display augmented reprogrammability of translational activities of two mRNA transcripts coding for rhα-Gal A and HLA-E proteins might provide a completely novel and powerful research tool for the panel of further studies. The objective of these future studies should be to multiply the tri-transgenic pigs with the aid of somatic cell nuclear transfer (SCNT)-based cloning for the purposes of both xenografting the porcine cutaneous bioprostheses and dermoplasty-mediated surgical treatments in human patients.

## 1. Introduction 

At the present stage of investigations in the fields of transplantation medicine and immunology, swine tissues and organs may be an alternative to their human counterparts. This is a highlight of research in an era of huge shortages of tissues and organs for allotransplantation, also taking into account a deficiency of allogeneic dermo-epidermal grafts in reconstructive medicine of the human integumentary system and dermoplasty-based therapies targeted at cutaneous/subcutaneous tissue engineering. The choice of pigs as donors of xenografts is not accidental. Firstly, anatomohistological and anatomotopographical biocompatibility of porcine tissues, organs and organ systems with their human counterparts determines, to a large extent, closely related physiological sufficiency of organs in these two mammalian species. Secondly, the genetic similarity between humans and domestic pigs is very high, reaching a level oscillating around 96%. Thirdly, *Sus scrofa domesticus* taxon is characterized by tremendously efficient outcomes noticed for species-specific fertility and prolificacy, which makes this livestock species relatively easy to breed [1,2,3,4,5]. Nonetheless, a lack of taxonomic consanguinity occurring between humans and pigs results in the appearance of interspecies immunophysiological incompatibility that largely limits and even prevents completely surgical treatments aimed at xenografting and xenogeneic tissue engineering. This interspecies immunological hindrance is triggered by the presence of a plasmalemma-anchored oligosaccharide moieties of glycoproteins and glycolipids, i.e., Galα(1,3)Galβ(1,4)GlcNAc-R that are designated as the α-Gal antigenic determinants or Galα1→3Gal epitopes on the surface of a vast majority of porcine cells (especially those forming vascular endothelium) [6]. The Galα1→3Gal epitope is common in mammals, but humans and apes have lost this structure through evolution [3,7]. Humans and apes produce natural antibodies against the Galα1→3Gal epitope. Unfortunately, the binding of these antibodies to the Galα1→3Gal epitope leads to hyperacute rejection (HAR) or acute humoral and cellular rejection of porcine xenografts [8,9,10]. The efforts undertaken to overcome the porcine→human immunological obstacle gave rise to the generation of transgenic pigs exhibiting a reduced or completely abrogated expression of the Galα1→3Gal epitopes [11]. The Galα1→3Gal epitopes can be removed from the surface of porcine cells by stably incorporating the h*FUT2* and h*GLA* gene constructs that is mediated by their intrapronuclear microinjection into porcine fertilized ova (zygotes) [4]. The h*FUT2* gene encodes the enzyme termed as α1,2-fucosyltransferase (H-transferase; α1,2-FT), which is responsible for the formation of the H structure, the core of the system of blood groups AB0 in humans. The action of α1,2-FT blocks the synthesis of the Galα1→3Gal epitope, while promoting the formation of the H structure, which is neutral for the human immune system [12]. The h*GLA* gene encodes human α-galactosidase A (α-Gal A), an enzyme that biocatalyzes the reaction directed to cleave terminal D-galactose residues from the Galα1→3Gal epitope. Deprivation of the Galα1→3Gal epitope of the terminal D-galactose molecules remarkably attenuates its xenoreactivity. This structure becomes undetectable by specific antibodies [13]. Another pivotal problem is the interspecies (porcine→human) immunological incompatibility that arises from phylogenetic divergence and subsequently brings about the species-specific variability at the level of major histocompatibility complex (MHC) proteins. To overcome this limitation, transgenic pigs expressing the *HLA-E* gene are indispensable. The effectiveness of this genetic modification has been proven by research confirming that the presence of human leukocyte antigen-E (HLA-E) molecules in porcine endothelial cells protects them against attack by human NK (natural killer) lymphocytes [14]. Pigs displaying the expression of the *HLA-E* transgene have been successfully created with the aid of intrapronuclear microinjection of the zygotes [15].

It is noteworthy that the applied genetic modification does not always result in a satisfactory (sufficiently high) level of expression of the transgenes integrated with the nuclear host genome. One of the ways to increase the expression of foreign (xenogeneic) genes introduced into the genomic DNA of recipient cells appears to be their epigenetic transformation applying non-specific inhibitors of histone deacetylases (HDACi). Our previous study [16] proved, for the first time, that the use of non-selective HDACi designated as trichostatin A (TSA) for the epigenomic modulation of porcine h*FUT2×*h*GLA* bi-transgenic adult cutaneous fibroblast cells (ACFCs) has contributed to improved reprogrammability followed by increased translational activities identified for mRNA transcripts synthesized from h*FUT2* and h*GLA* transgenes. Moreover, the action of TSA as a non-specific epigenetic modifier has been reflected in the augmented expression of pivotal genes coding for structural, enzymatic and other functional proteins indispensable for the biosynthesis and bioaccumulation of the Galα1→3Gal antigenic determinants. However, TSA has been shown to simultaneously enhance the expression of the incorporated h*FUT2*×h*GLA* gene constructs more strongly, so that the extent of Galα1→3Gal epitope silencing estimated for TSA-treated double-transgenic ACFCs was considerably higher than that noticed for TSA-untreated non-transgenic cells. Therefore, the TSA-mediated approach to epigenomically modulate the bi-genetically engineered variant of ACFC lines turned out to be beneficial, since it caused a remarkably diminished incidence of Galα1→3Gal epitopes in this ACFC type [16]. The molecular mechanisms underlying the capabilities of transgenes to epigenetically reprogram their transcriptional activities are complex. They involve amplifying the extent of lysine acetylation (i.e., hyperacetylation) within histones forming chromatin-derived nucleosomal cores. In our previously devised model of bi-transgenic ACFCs [16], hyperacetylation seemed to result from alleviation of biocatalytic functions of HDACs by TSA. A TSA-prompted decline in histone deacetylation may also affect other processes, such as the demethylation of DNA cytosine residues, incurring their intensification. A broad spectrum of non-specific HDACi, including TSA, and/or non-specific inhibitors of DNA methyltransferases (DNMTi) and/or selective inhibitors of histone methyltransferases (HMTi) have been formerly utilized in the strategies aimed to epigenomically modulate nuclear recipient oocytes [17,18,19], nuclear donor cells [20,21,22,23] and activated nuclear-transferred oocytes in pigs and other mammalian species [24,25,26]. These strategies have been developed to predominantly facilitate/refine the reprogramming of epigenomic memory and subsequently enhance the transcriptional activity of donor cell nuclear genomes in mammalian cloned embryos propagated by somatic cell nuclear transfer (SCNT) [27,28,29,30,31].

Taking into consideration all the aforementioned findings, h*FUT2*×h*GLA*×*HLA-E* tri-transgenic ACFC lines also needed to be tested separately by designing their models of the ex vivo migration and expansion under the conditions of TSA-dependent epigenetic transformation. In the current investigation, on the one hand, we have decided to unravel proteomic signatures related to relative abundances (RAs) estimated for HLA-E, rhα-Gal A and rhα1,2-FT in porcine triple-transgenic ACFCs undergoing TSA-assisted epigenomic modulation. On the other hand, the present study sought to decipher semi-quantitative profiles of α-Gal epitopes at the glycoprotein level. This research is the first to comprehensively assess the TSA-expedited enhancement of capabilities of transgene-encoded transcripts to epigenomically reprogram their translational activities in the ex vivo models elaborated to explore proteomic and glycoproteomic profiles in porcine h*FUT2*×h*GLA*×*HLA-E* tri-genetically engineered ACFC lines.

## 2. Results

### 2.1. Western Blot Analysis of the RA Pinpointed for HLA-E, rhα1,2-FT and rhα-Gal A Proteins in the Ex Vivo-Expanded Porcine Triple- and Non-Transgenic ACFCs Undergoing or Not Undergoing TSA-Mediated Epigenetic Transformation

**Figure 1:**
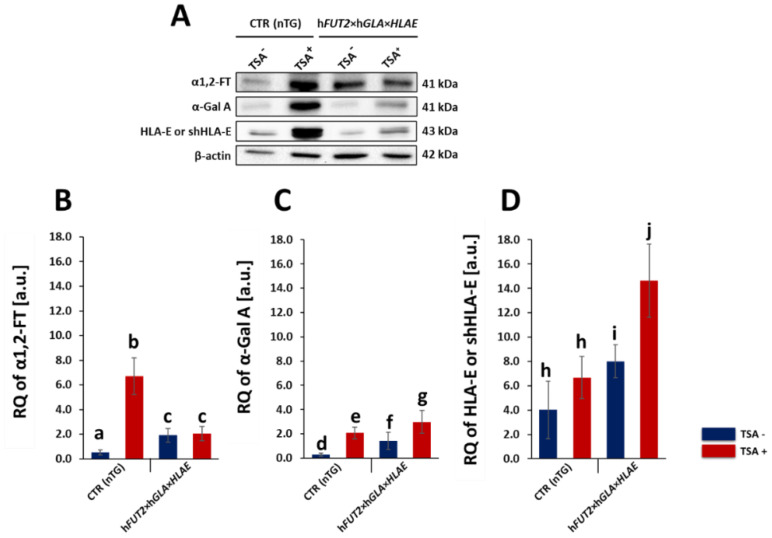
Western blot analysis of the relative expression of human α1,2-fucosyltransferase (α1,2-FT), α-galactosidase A (α-Gal A) and human leukocyte antigen-E (HLA-E) proteins in porcine triple-transgenic and non-transgenic ACFCs epigenetically transformed or not transformed with trichostatin A (TSA^+^ and TSA^−^, respectively). Representative blots of the expression of α1,2-FT, α-Gal A and HLA-E proteins in the ACFC samples derived from epigenomically modified (TSA^+^) and non-modified (TSA^−^) groups—panel (**A**). The samples stemming from non-transgenic pigs served as a control group (CTR nTG)—panel (**A**). β-Actin provided a loading control for all the analyzed samples. The results of relative expression (in arbitrary units) of α1,2-FT, α-Gal A and HLA-E or swine homolog of HLA-E (shHLA-E) are shown in panels (**B**–**D**), respectively. The relative optical density (ROD) from three separate analyses of at least three animals for each variant is expressed as mean. The bar graphs show the mean ± SEM. Statistics: one-way ANOVA and Newman–Keuls post hoc test. The bars marked with different letters vary significantly; values denoted as a-b, b-c, e-g: *p* < 0.01; a-c, d-e, e-g, f-g, h-i, h-j, i-j: *p* < 0.05.

In vitro proliferating ACFC lines exposed and not exposed to TSA (TSA^+^ and TSA^−^, respectively) were established from dermal explants that had been recovered post-mortem from either h*FUT2×*h*GLA×HLA-E* triple-transgenic pigs or their non-transgenic counterparts served as a control group (CTR nTG). Western blot analysis of total protein samples revealed the presence of rhα1,2-FT, rhα-Gal A and HLA-E proteins in all the tri-transgenic samples (Figure 1A). For the TSA^−^ control group, a weak positive signal stemming from all the analyzed proteins was noticed, but it was shown to be unremarkable. In contrast, Western blot analysis of total protein samples derived from TSA^+^ ACFCs confirmed the occurrence of clear positive signals for not only rhα1,2-FT but also rhα-Gal A and HLA-E. Signal intensities of analyzed proteins were normalized to β-actin, which was used as a loading control. The semi-quantitative analysis of Western blot strongly supported our findings. Indeed, the relative expression of the HLA-E and rhα-Gal A proteins has been proven to significantly increase (at least *p* < 0.05) in TSA-treated tri-genetically modified ACFCs as compared to the relevant samples originating from TSA-untreated 3×TG cells (Figure 1B–D). In turn, we did not observe significant differences in the relative expression of rhα1,2-FT between total protein samples derived from TSA^+^ and TSA^−^ triple-transgenic ACFCs. Interestingly, a clear positive signal was also identified for not only α1,2-FT and α-Gal A enzymes but also swine homolog of the HLA-E (shHLA-E) protein in TSA^+^ ACFCs stemming from CTR nTG pigs. This result has been confirmed both qualitatively and semi-quantitatively (at least *p* < 0.05) (Figure 1B–D). It is noteworthy that pigs are characterized by the lack of α-Gal A protein expression, which arises from species-specific silencing both alleles of p*GLA* gene. Taking this finding into consideration, the epigenetic alterations resulting from the conditions of either in vitro culture or TSA-assisted epigenomic modulation seem to trigger, to some extent, the onset of transcriptional and translational activities for p*GLA* alleles and their transcribed mRNAs in the ex vivo-expanded porcine CTR nTG ACFCs.

### 2.2. RT-qPCR-Mediated Confirmation of Stability in the Expression Profiles Pinpointed for hFUT2, hGLA and HLA-E mRNA Transcripts in the Ex Vivo-Expanded Porcine Triple-Transgenic ACFCs Subjected or Not Subjected to TSA-Assisted Epigenetic Transformation

**Figure 2:**
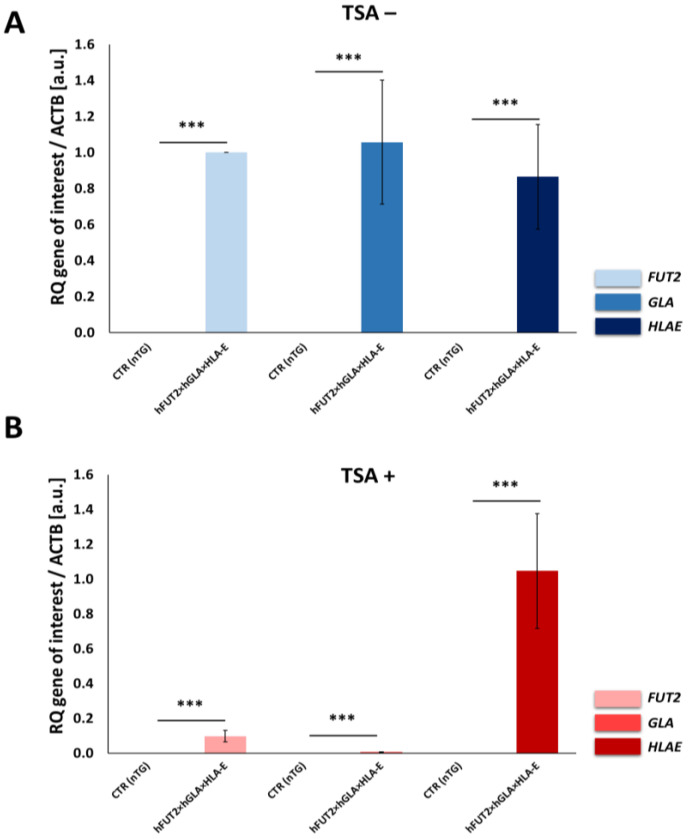
RT-qPCR analysis of the relative expression of human genes: h*FUT2*, h*GLA* and *HLA-E* in porcine triple-transgenic (3×TG) ACFCs epigenetically not transformed (**A**) or transformed (**B**) with trichostatin A (TSA− and TSA+, respectively). The p*ACTB* gene encoding porcine β-actin was used as endogenous reference gene. The bar graphs show the mean ± SEM. Statistics: one-way ANOVA and Newman–Keuls post hoc test. The bars marked with different letters vary significantly; values denoted as *** *p* < 0.001. The numbers of biological replicates (i.e., numbers of 3×TG and CTR nTG pigs serving as independent biological donors) ≥ 3. Number of technical replicates = 3 (within each biological replicate).

To ascertain the stability of transgene-encoded transcripts in porcine h*FUT2*×h*GLA*×*HLA-E* triple-transgenic ACFCs that either did or did not undergo TSA-dependent epigenetic transformation (TSA− and TSA+, respectively), the RT-qPCR analysis was performed to detect the quantitative expression profiles noticed for h*FUT2* mRNA, h*GLA* mRNA and *HLA-E* mRNA. The obtained results confirmed a significant upregulation of all three investigated human genes in porcine tri-transgenic ACFCs originating from the TSA− group (*p* < 0.001) (Figure 2A). In turn, estimating the relative quantities that were identified for h*FUT2*, h*GLA* and *HLA-E* transcripts in porcine triple-transgenic ACFCs stemming from the TSA+ group indicated a much smaller upward trend as compared to the control non-transgenic group (CTR nTG), but this augmentation also turned out to be statistically significant (*p* < 0.001) (Figure 2B).

### 2.3. Immunofluorescence Localization of HLA-E, rhα1,2-FT and rhα-Gal A in the Ex Vivo-Expanded Porcine Triple- and Non-Transgenic ACFCs Epigenetically Transformed or Not Transformed by TSA Treatment

**Figure 3:**
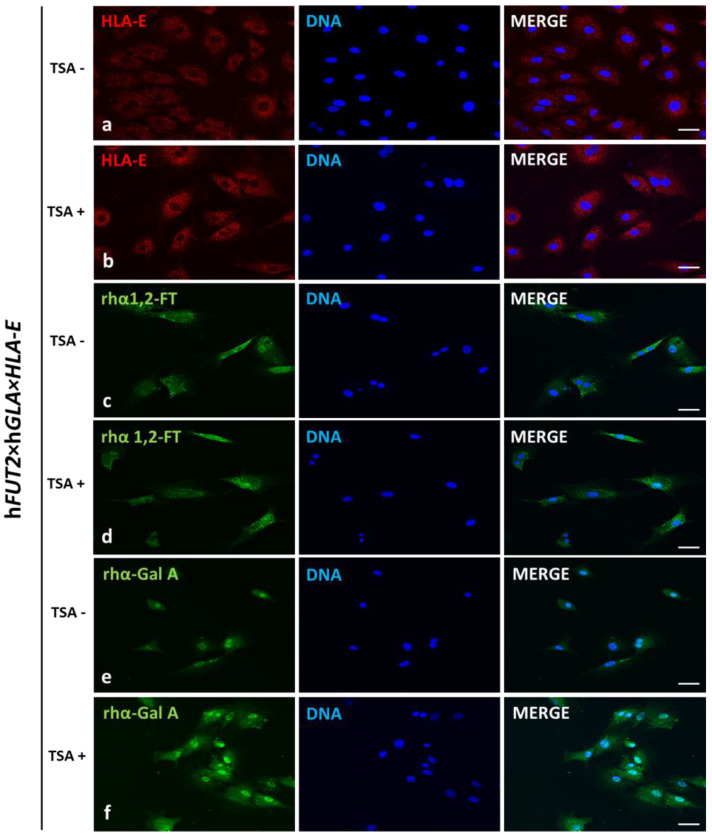
Immunofluorescence analysis of ex vivo-expanded porcine ACFCs epigenomically modulated (TSA+) (**b**,**d**,**f**) and not modulated with trichostatin A (TSA−) (**a**,**c**,**e**). Representative microphotographs of immunofluorescence localization of human leukocyte antigen-E (HLA-E; (**a**,**b**)), recombinant human α1,2-fucosyltransferase (rhα1,2-FT; (**c**,**d**)) and α-galactosidase A (rhα-Gal A; (**e**,**f**)) in ACFCs originating from h*FUT2*×h*GLA*×*HLA-E* triple-transgenic pigs. Immunofluorescent staining with Alexa Fluor 488- or Cy3-labelled secondary antibodies (green and red fluorescence, respectively) and DAPI counterstaining (blue fluorescence). Scale bars represent 100 μm. Immunoreaction was performed on ex vivo proliferating porcine ACFCs derived from at least three pigs of each experimental group. The immunofluorescence signal arisen from rhα1,2-FT was distributed in the perinuclear region of all the analyzed ACFCs from each variant. The MHC representative termed as HLA-E and rhα-Gal A enzyme were homogeneously located in whole cytoplasm of all the analyzed ACFCs. The TSA^+^ ACFCs were characterized by largely intensified signal estimated for both HLA-E and rhα-Gal A proteins (**b**,**f**), but not for rhα1,2-FT (**d**) as compared to their TSA^−^ cell counterparts (**a**,**c**,**e**).

**Figure 4:**
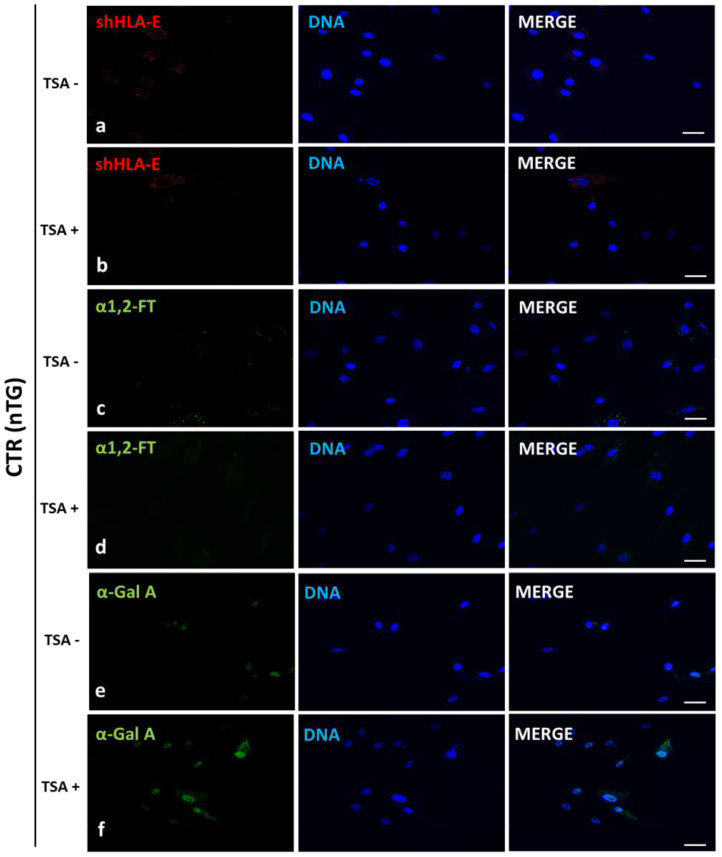
Immunofluorescence analysis of the ex vivo-expanded porcine ACFCs epigenomically modulated (TSA+) (**b**,**d**,**f**) and not modulated with trichostatin A (TSA−) (**a**,**c**,**e**). Representative microphotographs of immunofluorescence localization of swine homolog of human leukocyte antigen-E (shHLA-E; (**a**,**b**)), α1,2-fucosyltransferase (α1,2-FT; (**c**,**d**)) and α-galactosidase A (α-Gal A; (**e**,**f**)) in ACFCs stemming from non-transgenic pigs (CTR nTG). Immunofluorescent staining with Alexa Fluor 488- or Cy3-labelled secondary antibodies (green and red fluorescence, respectively) and DAPI counterstaining (blue fluorescence). Scale bars represent 100 μm. Immunoreaction was performed on ex vivo proliferating porcine ACFCs derived from at least three pigs of each experimental group. TSA^+^ ACFCs displayed more highly intensified signals identified for not only shHLA-E but also α1,2-FT and α-Gal A proteins in control non-transgenic (CTR nTG) ACFCs (**b**,**d**,**f**). No positive signals originating from either shHLA-E representative of MHC or α1,2-FT and α-Gal A enzymes were observed in the TSA^−^ CTR nTG group (**a**,**c**,**e**).

The localization of the HLA-E, rhα1,2-FT and rhα-Gal A proteins was examined by immunofluorescence staining of TSA-treated (TSA^+^) and untreated (TSA^−^) ACFCs derived from h*FUT2*×h*GLA*×*HLA-E* triple-transgenic (Figure 3) and non-transgenic (Figure 4) pigs used as a control group (CTR nTG).

The positive immunofluorescence signals descended from HLA-E and rhα-Gal A were found to be dispersed homogenously and intensively detectable in the whole cytoplasm of h*FUT2*×h*GLA*×*HLA-E* triple-transgenic ACFCs (Figure 3). In turn, the signal identified for rhα1,2-FT was mainly distributed in small perinuclear clusters in tri-genetically engineered ACFCs. Remarkably stronger immunofluorescence signals were observed for both HLA-E and rhα-Gal A proteins in TSA^+^ ACFCs (Figure 3b,f). Interestingly, no differences in rhα1,2-FT-derived immunofluorescence signal intensity took place between ACFCs exposed to TSA (TSA^+^) and their cell counterparts not exposed to this HDACi (TSA^−^) (Figure 3c,d). Moreover, a weak immunofluorescence signal stemming from all the analyzed proteins was noticed for control non-transgenic (CTR nTG) ACFCs subjected to TSA-based epigenetic transformation (Figure 4b,d,f). However, in TSA^−^ CTR nTG ACFCs, swine homolog of HLA-E and α1,2-FT enzyme were shown to be scarcely detectable by immunofluorescence, whereas no positive signal descended from intrinsic species-specific α-Gal A enzyme was identified (Figure 4).

### 2.4. Lectin Blotting Analysis of Galα1→3Gal Epitope Expression at the Protein Level in the Ex Vivo-Expanded Porcine Triple- and Non-Transgenic ACFCs Subjected or Not Subjected to TSA-Dependent Epigenetic Transformation

**Figure 5:**
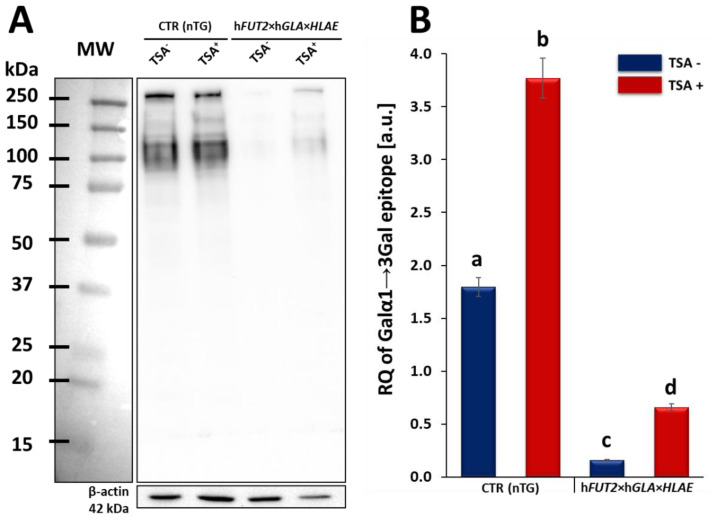
Lectin blot analysis of Galα1→3Gal epitope expression at the protein level in the ex vivo-expanded porcine h*FUT2*×h*GLA*×*HLA-E* tri-transgenic (3×TG) and control non-transgenic (CTR nTG) ACFCs epigenetically transformed (TSA^+^) or not transformed with trichostatin A (TSA^−^). (**A**) Representative lectin blots of the expression of Galα1→3Gal (α-Gal) epitopes in TSA^−^ and TSA^+^ ex vivo proliferating ACFCs established from dermal tissue explants stemming from CTR nTG and 3×TG pigs. MW indicates the molecular weight of protein standards (Precision Plus Protein^TM^ Dual Color Standards, Bio-Rad). Each band represents a glycosylated protein containing the Galα1→3Gal antigenic determinant. β-Actin provided a loading control for all the analyzed samples. (**B**) The semi-quantitative analysis of relative expression pinpointed for Galα1→3Gal epitopes (in arbitrary units). Relative optical density (ROD) from three separate analyses of at least three animals for each variant is expressed as mean. Graph bar shows the mean ± SEM. Statistics: one-way ANOVA and Newman–Keuls post hoc test. The bars marked with different letters vary significantly. Values denoted as a-b: *p* < 0.01; a-c, a-d, b-c, b-d, c-d: *p* < 0.05. It is worth highlighting that the semi-quantitative profile of Galα1→3Gal epitopes dwindled significantly in TSA-untransformed and transformed ACFCs stemming from h*FUT2*×h*GLA*×*HLA-E* triple-transgenic pigs as compared to the TSA-unexposed and exposed ACFCs originating from CTR nTG specimens. Nonetheless, the protein samples isolated from TSA-unmodulated 3×TG ACFCs exhibited the significantly lowest relative abundance (RA) noticed for Galα1→3Gal epitopes. Taking into consideration both tri-genetically modified and non-modified ACFCs, RAs estimated for Galα1→3Gal antigenic determinants increased significantly in protein samples derived from TSA^+^ cells as compared to those recognized for their protein counterparts extracted from TSA^−^ cells. However, the level of α-Gal epitopes pinpointed for TSA-modulated 3×TG ACFCs was still profoundly lower than the semi-quantitative profiles of α-Gal antigenic determinants observed in TSA-modulated and unmodulated CTR nTG ACFCs.

The expression profile of Galα1→3Gal epitopes at the total protein level was estimated by lectin blot analysis using horseradish peroxidase (HRP)-conjugated isolectin GS I-B_4_. The results of our study confirmed that significantly decreased expression of Galα1→3Gal epitopes was identified in h*FUT2*×h*GLA*×*HLA-E* tri-transgenic (3×TG) ACFCs as compared to their CTR nTG cell counterparts. In turn, for both groups (CTR nTG and 3×TG), the RA noticed for Galα1→3Gal antigenic determinants was proven to increase significantly in protein samples derived from ex vivo proliferating ACFCs epigenetically transformed with TSA (TSA^+^; Figure 5A). β-Actin served as a loading control protein. The semi-quantitative analysis of protein samples extracted from TSA-unmodulated (TSA^−^) and modulated (TSA^+^) tri-genetically modified ACFCs positively verified these observations. The significantly lowest RA estimated for Galα1→3Gal epitopes was identified in TSA-untransformed ACFCs originating from h*FUT2*×h*GLA*×*HLA-E* triple-transgenic pigs, indicating the occurrence of statistical variability in relation to the TSA^−^ CTR nTG (*p* < 0.05) and TSA^+^ CTR nTG groups (*p* < 0.05). The semi-quantitative profile of Galα1→3Gal epitopes in ACFCs stemming from the TSA^+^ 3×TG group was significantly higher (*p* < 0.05) than that pinpointed for ACFCs assigned to the TSA^−^ 3×TG group, but it was shown to be still lower than that recognized for both CTR nTG groups (*p* < 0.05) (Figure 5B).

## 3. Discussion

The use of swine tissues and organs seems to be a response to the current shortage of organs for allogeneic transplantation in humans, considering also a paucity of human dermo-integumentary allografts. However, due to hyperacute rejection, which is the main obstacle in pig-to-human xenotransplantation, genetically modified pigs need to be propagated and multiplied [4]. Currently, many methods of genetic modification are available. At first, they encompass the generation of homozygous pigs lacking the gene coding for α1,3-galactosyltransferase (α1,3-GT) for the purpose of depletion of anti-pig antibodies [11]. Secondly, removal of the Galα1→3Gal epitopes using genetically engineered enzymes is taken into account, ending at the production of pigs transgenic for some graft-protective proteins [32,33,34,35].

In our current study, we applied Western blotting and immunofluorescence staining with confocal microscopy to assess the effect of TSA-triggered epigenetic modulation both on the overexpression of HLA-E, rhα1,2-FT and rhα-Gal A proteins and on the RA of Galα1→3Gal antigenic determinants in porcine h*FUT2*×h*GLA*×*HLA-E* tri-transgenic ACFC lines as compared to their non-transgenic counterparts. Since genetically modified pigs have been successfully generated to avoid hyperacute rejection of tissue/organ xenografts [12,13,36], we have decided to conduct the next panel of research, in which we discuss this aspect in regard to the ex vivo models based on the mitotically stable triple-transgenic ACFCs undergoing TSA-prompted epigenetic transformation followed by profound examination of molecular signatures dependent on proteomic and glycoproteomic profiles. The results of Western blot and immunofluorescence analyses revealed considerable enhancements in the expression of rhα-Gal A and HLA-E but not rhα1,2-FT in TSA-modulated tri-genetically engineered ACFCs as compared to their cell counterparts not modulated by TSA treatment. Furthermore, immunofluorescence staining with antibodies against HLA-E, rhα1,2-FT and rhα-Gal A has provided strong evidence that TSA-assisted epigenetic transformation leads to a significant increase in the expression levels estimated for HLA-E and rhα-Gal A but not for rhα1,2-FT in triple-transgenic ACFC lines. Our previous study [16] has confirmed that the semi-quantitative profiles of both rhα-Gal A and rhα1,2-FT were characterized by remarkable augmentation in porcine h*FUT2*×h*GLA* bi-transgenic ACFCs subjected to TSA-mediated epigenomic modulation. In turn, lectin blotting analysis has demonstrated a similar decrease in the RAs pinpointed for Galα1→3Gal epitopes in TSA-modified triple-transgenic ACFCs as those recognized for α-Gal antigenic determinants in the TSA-modulated double-transgenic ACFC counterparts [16]. For those reasons, it can be assumed that the lack of a profound influence of TSA-induced epigenetic modification on the RA noticed for rhα1,2-FT in tri-genetically engineered ACFCs does not relieve the capability of this enzyme to abrogate the expression of the Galα1→3Gal epitopes. It is also possible that TSA-facilitated amelioration of the semi-quantitative profiles of rhα-Gal A alone had the remarkable effect of reducing the molecular levels of α-Gal antigenic determinants. Another study has proved that the biocatalytic activity of only human α-Gal A alone alleviated the relative abundance of Galα1→3Gal epitopes by 78%, and the co-expression of human α1,2-FT and α-Gal A enzymes diminished the quantitative profile of α-Gal antigenic determinants to a negligible level on the surface of porcine genetically modified aortic endothelial cells subjected to SV40-mediated immortalization [37]. The study by Wiater et al. [38], in which confocal microscopy and lectin blotting analyses were accomplished to molecularly evaluate the proteomic and glycoproteomic signatures occurring in liver-derived tissue explants originating from h*FUT2*×h*GLA* double-transgenic pigs, has shown that the co-expression of human α-Gal A and α1,2-FT enzymes has led to a decline in RAs of Galα1→3Gal epitopes by 62% (as has been proven on the basis of intensity of fluorescence) and by 47% (as has been proven on the basis of blotting), respectively. The failure to completely suppress the expression of Galα1→3Gal antigenic determinants in the above-indicated studies seems to arise from low molecular levels of rhα1,2-FT and rhα-Gal A enzymes. Therefore, the use of TSA-assisted epigenomic modulation of genetically engineered cells to enhance the relative expression of these enzymatic proteins appears to be a powerful tool suitable for the solution to this problem. However, taking into consideration the fact that the impacts of TSA-dependent epigenetic transformation of porcine tri-transgenic ACFCs have been exerted not only on the semi-quantitative profiles of rhα-Gal A and HLA-E proteins but also on the RAs recognized for Galα1→3Gal epitopes, these results are consistent with our previous study aimed at exploring the ex vivo models designed on porcine bi-transgenic ACFCs [16]. In turn, the present investigation has confirmed that TSA-evoked epigenomic modulation did not considerably affect the molecular signatures related to the semi-quantitative profiles identified for rhα1,2-FT enzyme. This finding can be scientifically justified by the overall existing expression of the aforementioned enzymatic protein in porcine tri-transgenic ACFCs, which turned out to be robustly lower than that noticed for porcine bi-transgenic ACFCs that have been proteomically and glycoproteomically evaluated in our former research [16]. Nevertheless, lectin blotting analysis revealed the considerably diminished relative abundance detected for Galα1→3Gal epitopes in both TSA-unmodulated and modulated porcine triple-transgenic ACFCs as compared to their non-transgenic cell counterparts.

The presence of α-Gal antigenic determinants is not the only hindrance in pig-to-human xenotransplantation. In this context, a pivotal role is also played by the immunological incompatibility between porcine and human tissues at the level of the MHC proteins. As a consequence, it was indispensable to generate and multiply the genetically modified pigs expressing HLA-E [15,36]. As one of the predominant members of the family involving MHC proteins, HLA-E has been allotted to a class Ib representatives of non-classical MHC molecules together with two additional members of this family, designated as HLA-F and HLA-G. Importantly, HLA-E occurs ubiquitously in all nucleated cells at relatively low levels, although it is expressed most abundantly in endothelial cells and various types of immune cells [39,40,41,42]. In addition, HLA-E is a major ligand for the inhibitory receptor of NK cells, which is termed as CD94/NKG2A [14,41,43], and the capacity of HLA-E to suppress macrophage-mediated cytotoxicity has been demonstrated [44,45]. Taking into account all these unique properties of HLA-E, our primary goal of great importance was to create such ex vivo h*FUT2*×h*GLA*×*HLA-E* tri-genetically engineered models of mitotically stable porcine ACFC lines, in which the semi-quantitative profiles estimated for HLA-E representative of MHC proteins were sustainably perpetuated at sufficiently high levels. For these reasons, the extensive efforts undertaken in the current research to perform the TSA-dependent epigenetic transformation of these triple-transgenic ACFCs have been proven to be a reliable and feasible strategy for the desirable achievement of a tremendously remarkable amelioration of relative abundance pinpointed for HLA-E protein. This TSA-expedited overexpression of HLA-E has been ascertained not only with respect to its molecular levels identified for TSA-untransformed 3×TG ACFCs but also, to an even more noticeable extent, with respect to semi-quantitatively profiling the swine homolog of HLA-E recognized for TSA-unmodulated and modulated non-transgenic cell counterparts.

Our current investigation provided, for the first time, clear evidence of a differentiable extent/advancement for the TSA-mediated augmentation of the capabilities of mRNA molecules transcribed from *HLA-E*, h*GLA* and h*FUT2* transgenes to epigenomically reprogram their translational activities in the ex vivo models designed on porcine tri-genetically engineered ACFCs. The TSA-dependent epigenetic transformation of h*FUT2*×h*GLA*×*HLA-E* triple-transgenic ACFCs has been also found to incur the enhancement of translational reprogrammability of mRNA transcripts synthesized not only from extrinsic (xenogeneic) genes but also from the own intrinsic genes within host nuclear DNA. Furthermore, no research has ever demonstrated the simultaneous overabundance of rhα-Gal A and HLA-E proteins followed by strong attenuation of semi-quantitative profiles identified for Galα1→3Gal antigenic determinants in porcine tri-transgenic ACFCs that have been epigenomically modulated by their exposure to a representative of potent non-selective HDACi, termed as trichostatin A. Nonetheless, TSA-assisted epigenetic transformation did not considerably affect the RAs of rhα-1,2-FT in 3×TG ACFC lines. Despite this fact, TSA-based epigenomic modulation did not relieve the biocatalytic capability of the rhα-1,2-FT enzyme to diminish the semi-quantitative profile of α-Gal epitopes. On the one hand, this finding can deliver an insightful interpretation of and meaningful justification for a sufficiently large and significant TSA-assisted impact on the expression levels estimated for rhα-Gal A. On the other hand, it can implement/incorporate new scientific knowledge and mechanistic insights into either the predominant role played by the enzymatic activity of rhα-Gal A or its possible synergistic cooperation with overabundant HLA-E proteins and strong inter-proteomic crosstalk between rhα-Gal A and HLA-E molecules. This intermolecular communication can be directed at pleiotropically and more efficiently prompting the tremendously alleviated expression of Galα1→3Gal antigenic determinants in h*FUT2*×h*GLA*×*HLA-E* triple-transgenic ACFCs. Furthermore, it is worth highlighting that, regardless of the molecular scenario by which the TSA-mediated epigenetic transformation of porcine tri-genetically modified ACFCs triggers the augmentation of the semi-quantitative profiles of α-Gal epitopes, the incidence of Galα1→3Gal antigenic determinants dwindled profoundly in both TSA-modulated and unmodulated h*FUT2*×h*GLA*×*HLA-E* tri-transgenic ACFCs exhibiting overexpression of rhα-Gal A and HLA-E proteins as compared to their non-transgenic cell counterparts.

In summary, these ex vivo models of mitotically stable and TSA-modulated h*FUT2*×h*GLA*×*HLA-E* triple-transgenic ACFCs, whose molecular signatures encompass not only downregulated glycoproteomic profiles of Galα1→3Gal antigenic determinants but also upregulated RAs of recombinant human immune enzymes (rhα-Gal A, rhα1,2-FT) and MHC representative (HLA-E), have been profoundly explored and might provide a completely new source of and powerful tool for highly reprogrammable (epigenomically dedifferentiable) nuclear donor cells for further studies. To the best of our knowledge, no research has ever been conducted to ascertain the suitability of such strongly reprogrammable h*FUT2*×h*GLA*×*HLA-E* tri-transgenic models of ACFC lines for future investigations targeted at multiplying tri-genetically modified pigs by SCNT-mediated cloning. The goals of these investigations might be focused on the use of the above-indicated tri-transgenic ACFC models, which are characterized by a genetically engineered diminished interspecies (porcine→human) immunological barrier, for the efforts undertaken to propagate and multiply cloned embryos, conceptuses and progeny by SCNT. These novel models of the ex vivo migrating and expanding triple-transgenic ACFC lines that exhibit TSA-facilitated epigenomic plasticity reflected in enhanced translational activities of desirable transgene-encoded transcripts might be a prerequisite for performing efficient preclinical and clinical trials aimed at the pig-to-human transplantation of dermo-epidermal xenografts conceptualized on the basis of porcine 3×TG ACFCs undergoing TSA-dependent epigenetic transformation. The swine cutaneous bioprostheses or substitutes of human skin, which are comprised of TSA-modulated porcine tri-genetically engineered ACFC lines, appear to be characterized by considerably diminished inter-species histophysiological and immunopathological incompatibility and shortened porcine→human immunogenetic distance. All these previously mentioned attributes might give rise to the amelioration of the capabilities of TSA-exposed porcine h*FUT2*×h*GLA*×*HLA-E* triple-transgenic ACFCs to cytophysiologically promote, perpetuate and expedite the kinetics of the ex vivo migration and proliferation of human cutaneous keratinocytes in hybrid (porcine→human) dermo-epidermal bioprostheses. Designing such skin bioprostheses based on porcine tri-transgenic ACFC xenografting seems to be profoundly reliable and feasible for preclinical and clinical studies focused on reconstructive surgery related to regenerative medicine treatments and dermoplasty-mediated tissue engineering of human integumentary system. A scarcity of allogeneic dermo-epidermal transplants in reconstructive and regenerative medicine triggers a necessity for creating transgenic animal models, including swine models, characterized by high histo- and anatomophysiological homology with the human dermo-integumentary system and genetically engineered attenuated interspecies immunological barrier. Such genetically modified swine models could provide biocompatible materials for developing desirable hybrid (porcine→human) or completely xenogeneic dermo-epidermal bioprostheses based on the ex vivo migration and expansion of multiple-transgenic ACFC lines. The latter could play a helping stimulatory function for the extracorporeal in situ proliferation of human and/or porcine epidermal keratinocytes. The aforementioned tools designed on swine ex vivo models of h*FUT2*×h*GLA*×*HLA-E* tri-transgenic ACFCs might be targeted at the future pre- and clinical trials undertaken to utilize the porcine tri-genetically engineered ACFC-based bioprostheses for the replacement or removal of: (1) hereditary anatomo- and histopathological changes within the human dermo-integumentary system; (2) malignant and non-malignant skin tumors; (3) surgical or burn skin wounds and scars or skin injuries; and (4) senescence-related alterations within the cutaneous and subcutaneous tissue compartments.

## 4. Materials and Methods

### 4.1. Establishment and TSA-Dependent Epigenetic Transformation of the Ex Vivo-Expanded ACFC Lines Stemming from Triple- and Non-Transgenic Pigs

The ACFC lines were established according to the protocols described in our previous studies [16,46]. In our current investigation, ACFCs derived from h*FUT2*×h*GLA*×*HLA-E* triple-transgenic pigs (*n* ≥ 3), which had been generated by the crossbreeding of h*FUT2*×h*GLA* double-transgenic pigs [47] with *HLA-E* single-transgenic specimens [36], were used. ACFCs originating from non-transgenic pigs served as a control group (CTR nTG; *n* ≥ 3). All animal procedures that were accomplished in the research by Hryhorowicz et al. [36] and Zeyland et al. [47] were conducted in accordance with the European Directive 2010/63/EU and approved by the Second Local Ethics Committee in Kraków, Poland (permission 1181/2015 from 21 May 2015). All ACFC lines were cultured in DMEM/F12 (1:1) (Sigma-Aldrich, St. Louis, MO, USA) enriched with 15% FBS (Sigma-Aldrich) and 1% penicillin/streptomycin cocktail (Sigma-Aldrich) in a CO_2_ incubator under stabilized conditions as follows: a temperature of +38.5 °C, 5% CO_2_ and relative humidity of air atmosphere ranging from 90 to 95%. For Western and Lectin blot analyses, cells were cultured in T-25 flasks of up to 2–3 passages, but for immunofluorescence, cells in the second passage were seeded onto sterile coverslips in 6-well plates. Immediately after the ex vivo-expanded ACFC lines reached approximately 85% of confluence, their epigenetic transformation was prompted by supplementation of the culture medium with 50 nM of TSA (Sigma-Aldrich). Both tri-transgenic and non-transgenic ACFCs were epigenomically modulated by treatment with TSA for 24 h. The effect of TSA-mediated epigenetic transformation on not only relative quantities of h*FUT2*, h*GLA* and *HLA-E* mRNA transcripts but also abundance profiles of adequate transgenically biosynthesized proteins and Galα1→3Gal epitopes was determined in the ex vivo-expanded ACFC lines originating from 3×TG (n ≥ 3) pigs. The ACFCs derived from non-transgenic (nTG; n ≥ 3) pigs provided TSA^+^ and TSA^−^ control groups. All the cell cultures were independently triplicated.

### 4.2. Total RNA Isolation, cDNA Synthesis and Reverse Transcription

Total RNA was extracted from either TSA-modulated or unmodulated h*FUT2*×h*GLA*×*HLA-E* triple-transgenic ACFCs and their non-transgenic cell counterparts. Total cellular RNA was isolated using the Total RNA Mini Plus Kit (A&A Biotechnology, Gdańsk, Poland) according to the manufacturer’s protocol. The quantity and quality of the total RNA were ascertained by measuring the absorbance at the detection wavelengths λ equal to 260 nm and 280 nm with a NanoDrop™ Lite Spectrophotometer (Thermo Scientific, Wilmington, DE, USA). Moreover, RNA samples were electrophoresed on a 1% (*w*/*v*) denaturing agarose gel to verify the RNA quality and stored frozen at −80 °C. First-strand cDNA was prepared by reverse transcription (RT) using 1 mg of total RNA, random primers and a High-Capacity cDNA Reverse Transcription Kit (Applied Biosystems, Foster City, CA, USA) according to the manufacturer’s protocol. The 20-mL total reaction volume contained random primers, dNTP mix, RNAse inhibitor and Multi Scribe Reverse Transcriptase. RT was performed in a T100 Thermal Cycler (Bio-Rad, Hercules, CA, USA) according to the following thermal profile: (1) 25 °C for 10 min, (2) 37 °C for 120 min and (3) 85 °C for 5 min. Genomic DNA amplification contamination was checked using control experiments, in which reverse transcriptase was omitted during the RT step. The samples were kept at −20 °C until further analysis.

### 4.3. Real-Time Quantitative Polymerase Chain Reaction (RT-qPCR)

The RT-qPCR was performed according to the manufacturer’s protocol. To quantitatively assess the transcriptional activities identified for each analyzed transgene (i.e., h*FUT2*, h*GLA* and *HLA-E*), the RT-qPCR reactions were successfully initiated and subsequently completed for each sample using a reaction mix prepared as follows: 1× SYBR Select Master Mix (Thermo Fisher Scientific), 2 μL of forward and reverse primers (1 μM each) and 4 μL of 20× diluted cDNA in a final volume of 15 μL. A no-RT control run was conducted with DNase-digested RNA to verify that the digestion was successful and sufficient for selected samples. The amplification protocol included an initial preheating at 50 °C for 2 min, initial denaturation at 95 °C for 10 min and 40 cycles of amplification (15 s at 95 °C and 60 s at 60 °C). A melting curve analysis was achieved at the end of each run. The RT-qPCR was carried out with a CFX96 Touch Real-Time PCR Detection System (Bio-Rad). The sequences of all the RT-qPCR primers are presented in Table 1.

**Table 1:** Primers used for RT-qPCR.

Gene	F/R	Primer Sequence (5′→3′)	T_m_ (°C)	Reference
h*FUT2*	F	ATGTCGGAGGAGCACGCGG	55.9	[12]
	R	CCACGGTGTAGCCTCCTGTCC	55.4	[12]
h*GLA*	F	GGGGAGGGGTTTTATGCGATGGAG	51.8	[13]
	R	CTGGCTCTTCCTGGCAGTCA	51.8	[13]
*HLA-E*	F	TTCCGAGTGAATCTGCGGAC	51.8	[36]
	R	AGGCGAACTGTTCATACCCG	53.8	[36]
p*ACTB*	F	CAAAGCCAACCGTGAGAAGA	53.8	[36]
	R	GTACCCCTCGTAGATGGGCA	53.0	[36]

Alterations in the quantitative profiles (i.e., relative quantities; RQs) noticed for adequate mRNA transcripts that had been triggered by the TSA-mediated epigenomic modulation of ex vivo-expanded h*FUT2*×h*GLA*×*HLA-E* triple-transgenic and non-transgenic ACFC lines were rendered as a ratio of target gene versus reference *ACTB* gene (coding for β-actin) in relation to expression in control samples using the method developed and optimized by Pfaffl [48] according to the following equation:

(1) $$Ratio=\frac{{\left(E_{target}\right)}^{nCt}\;Target^{\left(control-sample\right)}}{{\left(E_{reference}\right)}^{nCt}\;Reference^{\left(control-sample\right)}}$$

In the above-indicated algorithmic formulation, the mathematical designation *E* denotes the amplification efficiency, whereas the *nCt* symbol is assigned to the number of RT-qPCR cycles needed for the signal to exceed a predetermined threshold value. To minimize the error associated with the differences in the quantity of applied template, the analyses were run in triplicate (at least three biological replicates and three technical replicates within each biological replicate), and the results were averaged.

### 4.4. Total Protein Extraction and Western or Lectin Blot Analyses Accomplished to Ascertain the Semi-Quantitative Profiles Pinpointed for α1,2-FT, α-Gal A and HLA-E/shHLA-E Proteins or Galα1→3Gal Epitopes at the Glycoprotein Levels in the Ex Vivo-Expanded Triple- and Non-Transgenic ACFCs

Total protein was extracted from harvested ACFCs using radioimmunoprecipitation assay lysis buffer (RIPA buffer, Thermo Fisher Scientific, Waltham, MA, USA) containing 1% proteinase inhibitor cocktail (RIPA+PI; Bioshop Inc., Burlington, ON, Canada). After treatment with TSA, cells were washed twice with ice-cold PBS, then 300 µL of RIPA+PI was added per flask, and cells were harvested with cell scrapers. The samples were subsequently sonicated and centrifuged at 13,200 rpm for 15 min at +4 °C, and supernatant was collected. Protein concentration was estimated with microassay DC^TM^ Protein Assay (Bio-Rad Laboratories, Hercules, CA, USA) using bovine serum albumin (BSA) as a standard. Protein samples were stored at −80 °C for further analyses.

For sodium dodecyl-sulphate (SDS)-polyacrylamide gel electrophoresis (PAGE), protein samples were diluted in 2× Laemmli Sample Buffer (Bio-Rad Laboratories, Hercules, CA, USA) containing β-mercaptoethanol and denaturated at 99.5 °C per 5 min. Electrophoresis was performed with 5% stacking and 10% resolving polyacrylamide gels. Each lane was loaded with 20 µg of protein. Then, proteins were electro-transferred onto a poly(vinylidene fluoride) (PVDF) membrane (Immobilon-P; Merck, Darmstadt, Germany) at a constant amperage of 250 mA for 120 min.

For immunoblotting membranes, after several washes in TBS, they were blocked for 1 h in 5% non-fat milk in TBST (Tris buffer saline with 0.1% *v*/*v* Tween20; Bioshop Inc.). Subsequently, the membranes were rinsed several times in TBST and incubated overnight at +4 °C with the following primary antibodies: against HLA-E (diluted 1:1000 in TBST; mouse monoclonal antibodies, ab11820, Abcam, Cambridge, UK), human α1,2-FT (diluted 1:1000 in TBST; rabbit polyclonal antibodies, ab198712, Abcam) and human α-Gal A (diluted 1:1000 in TBST; rabbit polyclonal antibodies, PA5-27349, ThermoFisher Scientific, Waltham, MA, USA). β-Actin served as a loading control protein (diluted 1:2000 in TBST; mouse monoclonal antibodies, ab8224, Abcam). Then, membranes were washed several times in TBST and incubated with goat anti-rabbit or goat anti-mouse HRP-conjugated secondary antibodies (ThermoFisher Scientific, Waltham, MA, USA) at a dilution of 1:6000 in TBST for 1 h at room temperature.

For lectin blotting, membranes were blocked for 30 min in 1% BSA (Bioshop Inc.) in TBST. Then, membranes were washed three times in DPBS containing Ca^2+^/Mg^2+^ ions (Gibco^®^ ThermoFisher Scientific, Waltham, MA, USA) followed by TBS. In the next step, membranes were incubated overnight at +4 °C with isolectin GS I-B_4_ labelled with HRP (L5391, Sigma-Aldrich) diluted 1:2000 in DPBS. Finally, membranes were washed in TBS buffer.

For both Western blotting and lectin blotting, protein bands were detected by chemiluminescence using Clarity^TM^ Western ECL Blotting Substrate (Bio-Rad Laboratories, Hercules, CA, USA) and visualized with the ChemiDoc^TM^ XRS+ Imaging System (Bio-Rad Laboratories, Hercules, CA, USA). Protein bands were quantified using the Image Lab^TM^ 2.0 Software (Bio-Rad Laboratories, Hercules, CA, USA). Semi-quantitative analysis was performed for three separately repeated experiments for each control and experimental group and normalized on β-actin (reference protein)-related signal. This indicates that at least three biological replicates originated from at least three independent 3×TG and nTG pigs. Subsequently, each of these biological replicates was run in three technical replicates in either one Western blot assay or one lectin blot assay. Each analysis was calculated as follows:

(2) $$Relative\;expression=\frac{signal_{SAMPLE}}{signal_{\begin{array}{c} REFERENCE\;PROTEIN \end{array}}}\;$$

Subsequently, the results encompassing the relative expression of the HLA-E, rhα1,2-FT and rhα-Gal A enzymes were shown as a mean ± SEM.

### 4.5. Immunofluorescence Staining of the Ex Vivo-Expanded Triple- and Non-Transgenic ACFCs Epigenomically Modulated or Not Modulated by Their Exposure to TSA

Immediately after TSA-assisted epigenetic transformation, tri-genetically modified or non-modified ACFCs were washed with sterile PBS and fixed with 4% paraformaldehyde for 10 min at room temperature. After several washes in PBS, cells were blocked in 5% normal goat serum (NGS) in PBST (PBS containing 0.1% Triton X-100) for 30 min. Cells were then incubated overnight at +4 °C in a humidified chamber with the following primary antibodies (the same as those for Western blot) against HLA-E (diluted 1:300 in PBST), human α1,2-FT (diluted 1:150 in PBST) and human α-Gal A (diluted 1:200 in PBST). In the next step, cells were washed several times in PBST and incubated with goat anti-rabbit Alexa Fluor 488-conjugated or goat anti-mouse Cy3-conjugated secondary antibodies (diluted 1:500 in PBST; ThermoFisher Scientific, Waltham, MA, USA) for 1 h at room temperature. After final washes, ACFCs were mounted in Fluoroshield with 4′,6-diamidino-2-phenylindole (DAPI) mounting medium (F6057, Sigma-Aldrich, St. Louis, MO, USA). All the experiments were independently replicated thrice, which denotes that at least three biological replicates were collected from at least three independent 3×TG and nTG pigs. Each of these biological replicates was run in three technical replicates in a single immunoreaction assay. Fluorescently labelled ACFCs were examined as described in Section 4.6.

### 4.6. Confocal Microscope Analyses of the Ex Vivo-Expanded Triple- and Non-Transgenic ACFCs Undergoing or Not Undergoing TSA-Based Epigenetic Transformation

Fluorescently labelled cells were examined by confocal microscope Olympus FluoView 1200 on inverted stand IX83 (Olympus, Tokyo, Japan). A magnification objective of 40 times (NA = 0.95) was used, and diode laser (473 nm), diode laser (543 nm) and diode laser (405 nm) were applied to excite green (Alexa Fluor 488), red (Cy3) and blue (DAPI) fluorescence, respectively.

### 4.7. Statistical Analysis

For each TSA-transformed and -untransformed ACFC variant stemming from tri-genetically engineered (n ≥ 3) and non-engineered pigs (n ≥ 3) and for all analyses, three replications were performed. Quantitative data were expressed as the mean ± standard error of the mean (SEM) and examined using the Shapiro–Wilks *W* test for normality. Comparisons between the appropriate means were achieved by one-way analysis of variance (ANOVA) followed by Newman–Keuls post hoc test for multiple ranges. All statistical analyses were carried out using Statistica 13 Software (StatSoft Inc., Tulsa, OK, USA). Statistical significance was marked by letters at the appropriate charts. The bars that were marked with different letters vary significantly.

## 5. Conclusions

The current research creates, for the first time, strong scientific foundations that empirically justify the highly elevated epigenetic reprogrammability of porcine h*FUT2*×h*GLA*×*HLA-E* tri-genetically modified ACFC lines due to their TSA-dependent epigenomic modulation. This has been clearly proven by proteomically profiling in such completely novel ex vivo models designed on the triple-transgenic cells. The comprehensive deciphering of molecular signatures in these tri-transgenic ACFCs has remarkably confirmed the TSA-mediated augmentation of the RAs noticed for rhα-Gal A and HLA-E proteins, which arises from enhanced translational activities of mRNA transcripts undergoing stable synthesis from h*GLA* and *HLA-E* transgenes. The TSA-facilitated scenarios of increased expression levels identified for rhα-Gal A and HLA-E proteins simultaneously brought about a decline in the semi-quantitative profiles of Galα1→3Gal antigenic determinants recognized at the glycoprotein levels, as has been compared to the ex vivo non-transgenic models of ACFC lines. For these reasons, the present study has been targeted at thoroughly estimating the semi-quantitative profiles of α-Gal epitopes at the glycoprotein level, which have been successfully downregulated by the synergistic interplay and reciprocal cooperation of overabundant protein products translated from genetically engineered mRNA transcripts of overexpressed h*GLA*, *HLA-E* and h*FUT2* transgenes in tri-genetically modified ACFCs subjected to TSA-mediated epigenetic transformation.

## Data Availability

Not applicable.

## Abbreviations

3×TG	Triple-transgenic; tri-transgenic
ACFC	Adult cutaneous fibroblast cell
ACTB	β-Actin
α-Gal	Galα1→3Gal antigenic determinant (epitope)
α1,3-GT	α1,3-galactosyltransferase
CTR nTG	Control non-transgenic
DNMTi	Inhibitors of DNA methyltransferases
HAR	Hyperacute rejection
HDACi	Inhibitors of histone deacetylases
HLA-E	Human leukocyte antigen-E
MHC	Major histocompatibility complex
NK	Natural killer
RA	Relative abundance
rhα1,2-FT	Recombinant human α1,2-fucosyltransferase
rhα-Gal A	Recombinant human α-galactosidase A
RT-qPCR	Reverse transcription-quantitative polymerase chain reaction
SCNT	Somatic cell nuclear transfer
shHLA-E	Swine homolog of human leukocyte antigen-E
TSA	Trichostatin A
